# CD8^+ ^T cell activation predominate early immune responses to hypercholesterolemia in *Apoe*^-/- ^mice

**DOI:** 10.1186/1471-2172-11-58

**Published:** 2010-12-02

**Authors:** Daniel Kolbus, Ornélia H Ramos, Katarina E Berg, Josefin Persson, Maria Wigren, Harry Björkbacka, Gunilla Nordin Fredrikson, Jan Nilsson

**Affiliations:** 1 Department of Clinical Sciences, Skane University Hospital Malmö, Lund University, Lund, Sweden; 2 Faculty of Health and Society, Malmö University, Malmö, Sweden

## Abstract

**Background:**

It is well established that adaptive immune responses induced by hypercholesterolemia play an important role in the development of atherosclerosis, but the pathways involved remain to be fully characterized. In the present study we assessed immune responses to hypercholesterolemia induced by feeding *Apoe^-/- ^*mice a high-fat diet for 4 or 8 weeks.

**Results:**

The primary immune response in lymph nodes draining the aortic root was an increased expression of interferon (IFN)-γ in CD8^+^CD28^+ ^T cells, while an activation of IFN-γ expression in CD4^+ ^T cells was observed only after 8 weeks of high-fat diet. Contrarily, spleen CD4^+ ^T cells responded with a higher expression of IL-10. Spleen CD8^+ ^T cells expressed both IFN-γ and IL-10 and showed enhanced proliferation when exposed to Concanavalin A. Plasma levels of IgG and IgM against oxidized LDL did not change, but the level of apolipoprotein B/IgM immune complexes was increased.

**Conclusion:**

Hypercholesterolemia leads to unopposed activation of Th1 immune responses in lymph nodes draining atherosclerotic lesions, whereas Th1 activation in the spleen is balanced by a concomitant activation of Th2 cells. The activation of CD8^+ ^T cells implies that hypercholesterolemia is associated with formation of cell autoantigens.

## Background

Both innate and adaptive immune responses contribute to the arterial inflammation that characterizes atherosclerosis [1-3]. Mice lacking critical components of innate immunity, such as the Toll-like receptor (TLR) 2 and 4 and the TLR signaling protein MyD88, develop less atherosclerosis, indicating the involvement of pro-atherogenic endogenous TLR ligands [4-6]. The role of adaptive immunity in atherosclerosis is more complex. While there is strong evidence that Th1 cells aggravate atherosclerosis [7-9] the possible influence of Th2 cells is less clear [10,11]. Regulatory T cells (Tregs) [12,13] and B cells [14] appear to have protective functions. A common feature of the studies that have revealed these associations is that atherosclerosis has been induced by hypercholesterolemia. Accordingly, it is likely that the immune responses that contributed to atherosclerosis development in these animals have been activated by ligands and antigens generated by hypercholesterolemia. The exact identity of these factors, as well as their mode of action, remains to be fully characterized. Attention has focused on the role of oxidized low-density lipoprotein (LDL) [15]. LDL particles become oxidized by various enzymes and oxygen metabolites when entrapped in the extra cellular matrix of the artery wall [16]. Oxidized LDL is targeted by both IgM and IgG autoantibodies [17] and as much as 10% of the T cells present in atherosclerotic plaques are specific for antigens formed in oxidized LDL [18]. T cells specific for oxidized LDL are also present in the circulation [19] and transfer of CD4^+ ^T cells isolated from mice immunized with aldehyde-modified LDL results in a more aggressive development of atherosclerosis [20] providing direct evidence for a pathogenic role of adaptive immunity against modified LDL in the disease process. Based on this knowledge attempts have been made to develop immunomodulatory therapy for prevention of cardiovascular disease and pilot vaccines containing apolipoprotein B (apo B) antigens have been shown to significantly reduce atherosclerosis in apolipoprotein E deficient (*Apoe^-/-^*) mice [21-23]. A limiting factor in the development of these therapies has been the poor understanding of the immune pathways activated by hypercholesterolemia [24]. In the present studies we aimed to address this issue by characterizing the induction of adaptive immunity to hypercholesterolemia both systemically and in regional lymph nodes draining lesion-prone areas of the aorta. We used *Apoe^-/- ^*mice in which a primary immune response to hypercholesterolemia-associated antigens, such as oxidized LDL, develops spontaneously [25]. To increase the antigen load we fed the mice a high-fat diet.

## Methods

### Animals

Female apolipoprotein E deficient mice on a C57BL/6 background were purchased from Taconic, USA. The animals were kept under controlled laboratory conditions in individually ventilated cages and food and water were provided *ad libitum*. All mice received chow diet until the age of 10 weeks. One group (n = 27) was then transferred to a high fat diet with 0.15% cholesterol and 21% fat (Lantmännen, Sweden) while the other group (n = 24) remained on chow diet. Mice were killed 4 weeks (high fat diet fed; n = 14, chow fed; n = 12) and 8 weeks (high fat diet fed; n = 13, chow fed; n = 12) after diet change, tissues were harvested and analyzed. The experiments were approved by the Animal Care and Use Committee of Lund University.

### Analysis of plaque autoantibody, apolipoprotein B and oxidized LDL content

The heart and proximal part of the aortic arch was embedded in OCT (Tissue-Tek). Frozen sections of 10 μm were collected from the subvalvular region. For detection of IgG or IgM, slides were fixed in ice-cold acetone for 5 min and blocked with 10% mouse serum in PBS for 30 min. To detect IgM and IgG autoantibodies, slides were incubated with biotinylated anti-mouse IgM or IgG antibodies (Vector Laboratories) for 50 min at room temperature. For the detection of apo B and oxidized LDL, slides were fixed in ice-cold acetone for 5 min and blocked with 10% goat serum in PBS for 50 minutes and incubated overnight at 4°C with primary rabbit anti-mouse apo B (Abcam) or anti-human malondialdehyde (MDA)-apo B peptide (2D03, Bioinvent, Sweden) antibodies diluted in 10% goat serum in PBS. A biotinylated goat anti-rabbit IgG antibody (Vector Laboratories) diluted in PBS was used as secondary antibody. The reaction products were visualized with Vectastain ABC elite kit (Vector Laboratories) using DAB as substrate (Vector Laboratories). Slides were counter-stained with hematoxylin and omission of primary or secondary antibodies were used as controls. Immune-stained areas were quantified with BioPix iQ 2.0 software (Biopix, Sweden).

### Cell preparation and flow cytometry

Mice were fasted for at least 3 hours before anesthetization by intraperitoneal injection of Xylazine (Rompun, Bayer Health care), and Ketamine (Ketalar, Pfizer) followed by euthanization by heart puncture. The spleen, mediastinal, brachial, axial, renal, iliac, sacral lymph nodes and thymus were removed and meshed through a cell strainer (70 μm, BD Bioscience). The single cell suspension of the lymph nodes and thymus were washed in RPMI medium (Gibco) and resuspended in complete medium (RPMI 1640 supplemented with 10% FCS, 1% Sodium pyruvate, 1% Hepes, 1% Penicillin/Streptomycin, 1% L-Glutamine and 0.1% β mercaptoethanol [Gibco]). Splenocytes were pelleted and resuspended in red blood cell lysing buffer (Sigma) for 2 minutes at room temperature to remove erythrocytes. Cells were washed and distributed in a 96 well round bottom plate (Sarstedt) at a density of 2 × 10^6 ^cells/ml and incubated overnight in a humidified chamber at 37°C and 5% CO_2_. Day 2, the cells were incubated with 5 μg/ml brefeldin A (eBioscience), 50 ng/ml PMA and 1 μg/ml ionomycin (all from Sigma) for 4 hours. Cells were incubated with a Fc-receptor blocking antibody (FcR;CD16/32; clone 93, Biolegend) for 5 minutes followed by incubation with CD28-PE/Cy5 (clone 37.51), CD4-PE/Cy7 (GK1.5), CD25-APC (PC61) and CD8-APC/Cy7 (53-6.7 all Biolegend) at 4°C for 30 minutes. The cells were resuspended in Fix/Perm solution (eBioscience), washed with permeabilization buffer (eBioscience) and blocked with FcR block prior to incubation with FoxP3-PB (clone FJK-16 S, eBioscience), IFN-γ- FITC (clone XMG1.2, Biolegend), IL-10-PE (clone JES5-16E3, Biolegend) for 30 minutes at 4°C. Cells were washed with permeabilization buffer and resuspended in FC buffer (1% fetal calf serum [Gibco] and 0.5 mM EDTA in phosphate buffered saline). Measurements were performed using a Cyan ADP (Beckman Coulter), analyses were performed using Summit (Beckman Coulter, version 4.3) and gating was adjusted using a negative control staining.

### Proliferation assay

Splenocytes (5 × 10^6^) were centrifuged and resuspended in 1 ml PBS. The cells were incubated with 10 μL Carboxyfluorescerein Succinimidyl ester (CFSE; Invitrogen, diluted 1:10 in PBS) for 5 minutes. The reaction was stopped with addition of 1 ml FCS, cells were washed with PBS and resuspended in 5 ml complete medium (see above). Cells (1 × 10^6^/ml) were transferred to a 96 well round bottom plate (Sarstedt) and incubated in presence or absence of 2.5 μg/ml Concanavalin A (ConA; Sigma) for 4 days at 37°C and 5% CO_2_. Cells were stained with FcR block (CD16/32; Biolegend) prior to staining with CD3-PE/Cy5 (clone 145-2C11), CD4-PE/Cy7 and CD8a-APC/Cy7 (all Biolegend). Stained cells were acquired in a gate comprising 50 000 cells and statistics were calculated on the number of CD4^+ ^and CD8^+ ^cells that had proliferated more than one time. Cells were acquired using a Cyan ADP (Beckman Coulter) and analyses were performed using Summit (Beckman Coulter, version 4.3). For cytokine analysis 5 × 10^5 ^splenocytes were cultured alone or with 2.5 μg/ml Con A for 72 hours and cytokine concentrations were measured in the cell culture supernatant using a Th1/Th2 9-plex (Meso Scale Discovery) according to the instructions of the manufacturer. The lower detection limit in this assay was 5 pg/ml.

### Plasma oxidized-LDL specific antibodies and Apolipoprotein B immune complex analysis

Plasma oxidized-LDL-specific antibodies were analyzed in Cu^2+ ^oxidized (10 μg/mL in PBS) or MDA-p210 peptide (KTTKQ SFDLS VKAQY KKNKH; 10 μg/mL in PBS) coated microtiter plates [26]. Plasma antibodies were detected using antibodies recognizing mouse IgG, IgM (Jackson ImmunoResearch), IgG1 (557272, BD Pharmingen) and IgG2a (553389, BD Pharmingen; cross reacts with IgG2c). In the apolipoprotein B immune complex assay, microtiter plates were coated with rabbit anti-apolipoprotein B antibody (ABcam), and the plasma apo B containing immune complexes were detected with the anti-mouse IgG1 and IgG2a detection antibodies described above.

### Analysis of cholesterol and triglyceride content

Total plasma cholesterol and plasma triglycerides were quantified with colorimetric assays, Infinity™ Cholesterol and Triglyceride (Thermo Electron).

### Statistical analysis

Analysis of data was performed using unpaired t test or Mann Whitney test for skewed data. Data are presented as mean ± standard deviation. Analysis was performed using GraphPad Prism 5.01 (Graphpad software) and a level of *P *< 0.05 was considered significant.

## Results

### Effect of diet on plasma lipids and atherosclerosis

**Figure 1:**
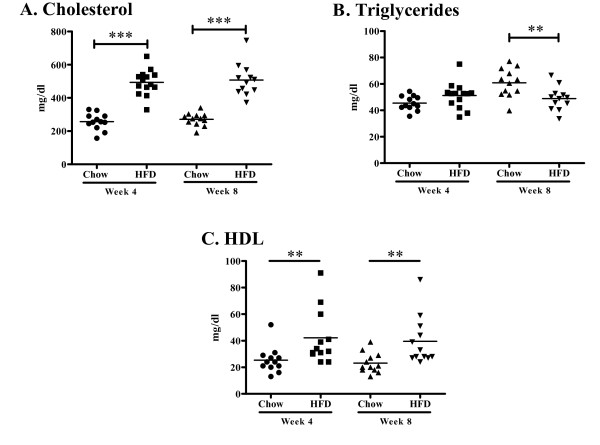
**Plasma cholesterol and triglyceride levels in high fat diet (HFD) and chow diet fed mice**. (A) Plasma concentration of cholesterol in mice fed a high-fat diet (HFD) and mice fed a chow diet at 4 and 8 weeks of respective diet. (B) Plasma triglyceride concentration at week 4 and 8 weeks in HFD fed mice and chow fed mice. (C) Plasma HDL concentration at week 4 and 8 weeks in HFD fed mice and chow fed mice. **P < 0.01, ***P < 0.001.

**Figure 2:**
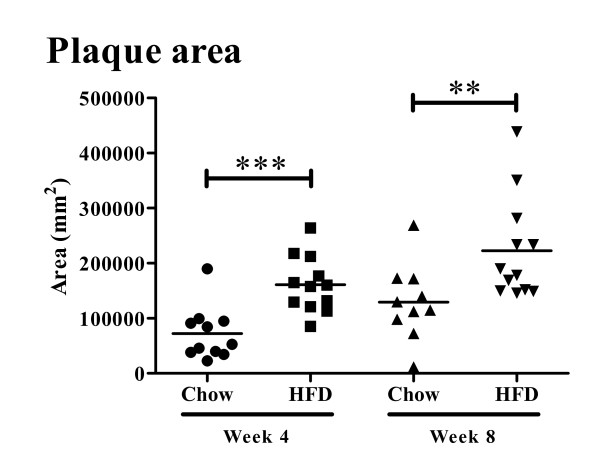
**Plaque area in aortic root**. Quantification of plaque area in the aortic root of chow and high-fat diet (HFD) fed mice at 4 and 8 weeks. **P < 0.01, ***P < 0.001.

**Figure 3:**
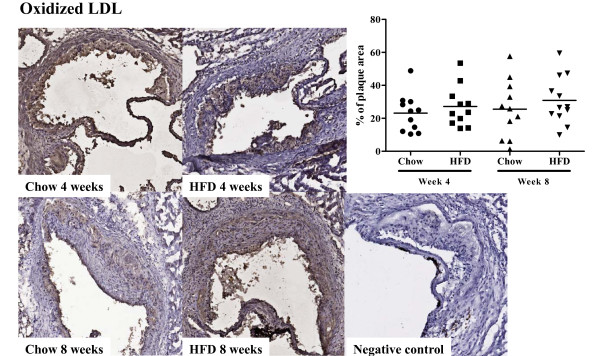
**Oxidized LDL deposition in the aortic root**. Immunohistochemical staining and quantification of the fraction of oxidized LDL (oxLDL) in aortic root sections from mice fed chow or high fat diet at 4 and 8 weeks respectively.

**Figure 4:**
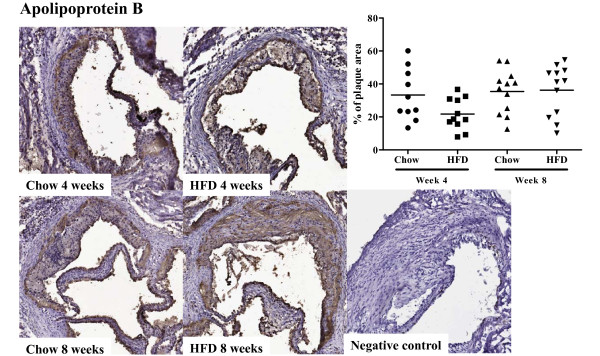
**Apolipoprotein B deposition in the aortic root**. Immunohistochemical staining and quantification of the fraction of apolipoprotein B (ApoB) in aortic root sections from mice fed chow or high fat diet at 4 and 8 weeks respectively.

*Apoe^-/- ^*mice fed high fat diet had twice as high plasma cholesterol levels as *Apoe^-/- ^*mice remaining on chow diet at week 4 (493.6 ± 77.4 vs. 256.7 ± 50.7 mg/dl, *P *< 0.001, Figure 1A) and week 8 (508.0 ± 98.0 vs. 270.9 ± 38.8 mg/dl, *P *< 0.001, Figure 1A). Similarly, plasma HDL concentration was higher in mice fed high fat diet (42.2 ± 20.6 vs. 25.4 ± 9.9 mg/dl, *P *< 0.01 at week 4 and 39.5 ± 18.3 vs. 23.2 ± 7.6 mg/dl at, *P *< 0.01 at week 8. Figure 1C). Triglyceride levels did not differ between the groups at 4 weeks (51.2 ± 9.8 (high fat diet) vs. 45.5 ± 5.5 (chow) mg/dl, but were significantly lower in the high-fat group at 8 weeks (48.9 ± 8.9 vs. 60.9 ± 10.8 mg/dl, *P *< 0.01, Figure 1B). Four weeks of high fat diet generated a more than two-fold increase in aortic root atherosclerotic plaque area as compared with chow diet (1.6 ± 0.51 × 10^5 ^vs. 0.72 ± 0.47 × 10^5 ^μm^2 ^(*P *< 0.001, Figure 2). The plaque area remained significantly increased in the high-fat group after 8 weeks of diet (2.2 ± 0.93 × 10^5 ^vs. 1.3 ± 0.68 × 10^5 ^μm^2^; *P *< 0.01, Figure 2) but the rate of progression between 4 and 8 weeks was approximately the same in both groups. The fraction of the plaque demonstrating positive immune-staining for oxidized LDL was similar in both groups and for both time points (27.1 ± 12.3 vs. 23.1 ± 11.5% at 4 weeks and 30.9 ± 14.4 vs. 25.5 ± 17.5% at 8 weeks, Figure 3). The same was true for apo B immunoreactivity (21.8 ± 9.6 vs. 33.3 ± 15.6% at 4 weeks and 36.2 ± 15.3 vs. 35.4 ± 13.2% at 8 weeks, Figure 4). These findings suggest that the effect of a more severe hypercholesterolemia on plaque growth was restricted to the first 4 weeks of diet.

### T cell activation

**Figure 5:**
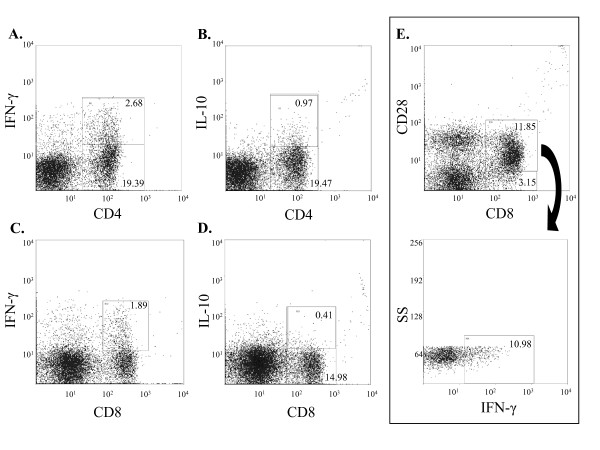
**Gating strategy at analysis of T cells**. Representative figures depicting gating strategy for (A) CD4^+^IFN-γ^+^, (B) CD4^+^IL-10^+^, (C) CD8^+^IFN-γ^+^, (D) CD8^+^IL-10^+ ^and (E) CD8^+^CD28^+ ^IFN-γ^+ ^cell populations. SS; side scatter.

To determine if hypercholesterolemia and plaque growth were associated with activation of adaptive immunity we analyzed T cell activation in the spleen and the mediastinal lymph nodes draining the aortic root, as well as brachial, axial, renal, iliac, and sacral lymph nodes, thymus and the circulation. The analysis focused on IFN-γ and IL-10 secreted by polyclonally activated CD4^+ ^and CD8^+ ^T cells as depicted in Figure 5. We detected a diet-dependent immune response in spleen and mediastinal lymph nodes but not in any of the other tissues analyzed (data not shown) which prompted us to focus the analysis on the two prior tissues.

### T cell activation in plaque draining lymph nodes

**Figure 6:**
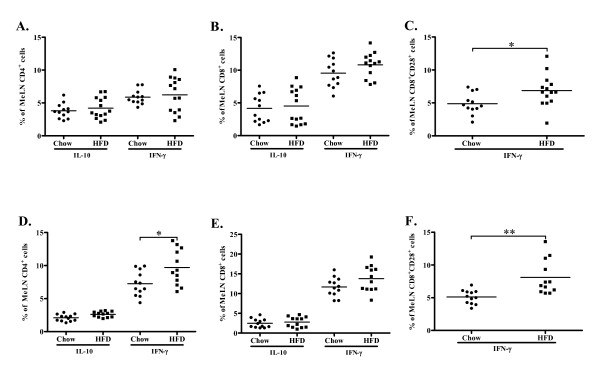
**Expression of IFN-γ and IL-10 in CD4^+ ^and CD8^+ ^cells in mediastinal lymph nodes (MeLN)**. Fig. A-C depicts MeLN cells at 4 weeks and fig. D-F depicts MeLN cells at 8 weeks. There was no difference in IL-10 or IFN-**γ **production in (A) CD4^+ ^or (B) CD8^+ ^cells between mice fed a high fat diet (HFD) and a chow diet. (C) HFD-fed mice expressed a higher fraction of IFN-**γ **producing CD8^+^CD28^+ ^cells compared to mice fed chow diet. (D) Increased IFN-**γ **production of CD4^+ ^cells of high fat diet (HFD) fed mice compared to CD4^+ ^cells from mice fed a chow diet. (E) No difference in the relative size of CD8^+^IFN-γ^+ ^and CD8^+^IL-10^+ ^cells between mice fed HFD and chow diet. (F) Increased IFN-**γ **production in CD8^+^CD28^+ ^cells in HFD-fed mice compared to mice fed chow diet. *P < 0.05, **P < 0.01.

**Figure 7:**
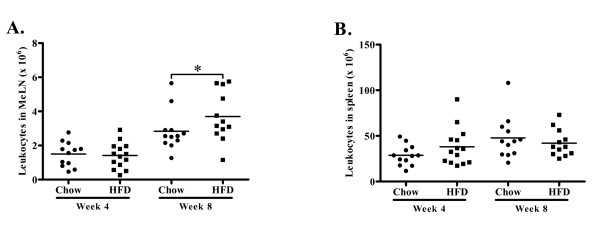
**Total leukocyte counts**. (A) There was no difference in MeLN total leukocyte number in mice receiving a high fat diet (HFD) compared to mice fed a chow diet at 4 weeks of diet but increased leukocyte number at 8 weeks of HFD compared to chow diet. (B) There was no difference in spleen total leukocyte number between mice receiving HFD compared to mice fed chow diet at 4 and 8 weeks of diet. *P < 0.05.

In spite of a markedly enhanced plaque formation there was no evidence for increased activation of CD4^+ ^cells in draining lymph nodes after 4 weeks of high-fat diet (Figure 6A). The fraction of CD4^+ ^expressing IFN-γ and IL-10 was approximately equal. There was also no effect on CD4^+^CD25^+^FoxP3^+ ^natural Tregs (8.1 ± 1.5% vs. 10.7 ± 8.2% of CD4^+ ^cells, non significant, n.s.), CD4^+^CD28^+ ^IFN-γ^+ ^T cells (12.6 ± 6.1% vs. 16.4 ± 6.5% of CD4^+ ^cells, n.s.) or CD8^+ ^T cells (Figure 6B). However, when the analysis was restricted to the CD28^+ ^population of CD8^+ ^T cells an increased expression of IFN-γ was observed in the high-fat group (Figure 6C). There was no difference in total mediastinal lymph node cell numbers between the chow and high-fat groups at 4 weeks (Figure 7A).

At the 8 week time point clear signs of CD4^+ ^cell activation could be observed in the mediastinal lymph nodes. The fraction of CD4^+ ^cells expressing IFN-γ was three-fold higher than the fraction of CD4^+ ^cells expressing IL-10 and the CD4^+ ^expression of IFN-γ was higher in the high-fat group (Figure 6D). The expression of IFN-γ remained elevated in CD8^+^CD28^+ ^T cells (Figure 6F), while no changes in the population of CD4^+^CD25^+^FoxP3^+ ^(8.6 ± 2.1% vs. 7.2 ± 1.6% of CD4^+ ^cells, n.s.), CD4^+^CD28^+ ^IFN-γ^+ ^(31.7 ± 4.5% vs. 30.1 ± 4.3% of CD4^+ ^cells, n.s.) or CD8^+ ^T cells were detected (Figure 6E). The total mediastinal lymph node cell numbers was increased as compared to the 4 week time point, and there was also a further increase in cell numbers in the mice given high-fat diet (Figure 7A).

### T cell activation in the spleen

**Figure 8:**
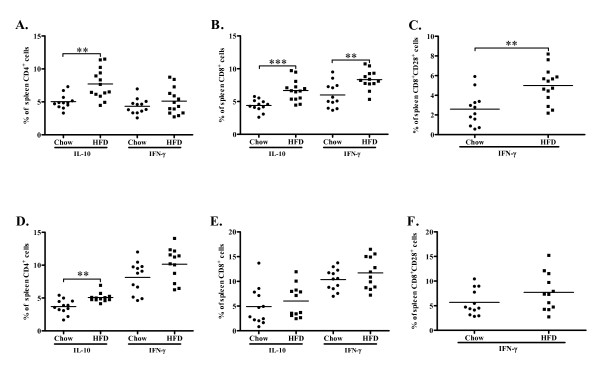
**Expression of IFN-γ and IL-10 in CD4^+ ^and CD8^+ ^cells in spleen**. Fig. A-C depicts splenocytes at 4 weeks and fig. D-F depicts splenocytes at 8 weeks. (A) Increased expression of IL-10 but not IFN-**γ **in CD4^+ ^cells in high fat diet (HFD) fed mice compared to mice fed chow diet. (B) Increased expression of IL-10 and IFN-**γ **in CD8^+ ^and (C) IFN-γ in CD8^+^CD28^+ ^cells in HFD-fed mice compared to chow fed mice. (D) Increased production of IL-10 in CD4^+ ^cells but not (E) CD8^+ ^cells and no difference in IFN-**γ **production in (D) CD4^+^, (E) CD8^+ ^cells or (F) CD8^+^CD28^+ ^cells in high fat diet (HFD) fed mice compared to mice fed chow diet. **P < 0.01, ***P < 0.001.

**Figure 9:**
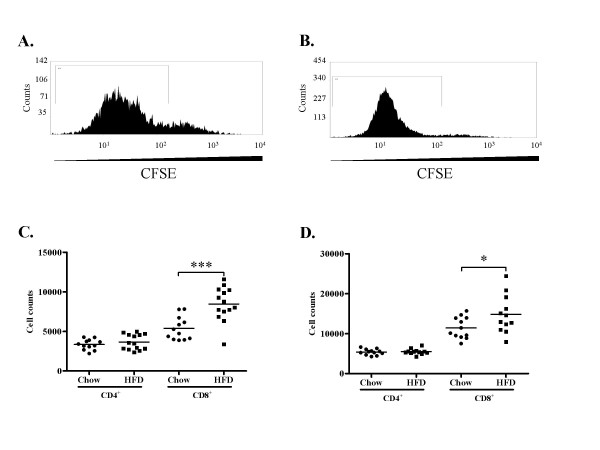
**Proliferation of CD4^+ ^and CD8^+ ^cells in spleen**. Representative histograms of (A) CD4^+^CFSE^+ ^and (B) CD8^+^CFSE^+ ^cells. Gates depict cells proliferating >1 time. (C-D) Proliferation of CD4^+ ^and CD8^+ ^T cells in mice fed chow or high fat diet (HFD) at 4 (C) and 8 (D) weeks of diet. *P < 0.05, ***P < 0.001.

Next we analyzed the systemic immune responses to hypercholesterolemia. After 4 weeks of high-fat diet the expression of IL-10 was increased in spleen CD4^+ ^cells, whereas no effect on CD4^+^IFN-γ^+ ^(Figure 8A), CD4^+^CD28^+ ^IFN-γ expression (9.2 ± 3.5% vs. 11.3 ± 4.8% of CD4^+ ^cells, n.s.) or CD4^+^CD25^+^FoxP3^+ ^(2.5 ± 0.8% vs. 3.4 ± 2.9% of CD4^+ ^cells, n.s.) populations were observed. The expression of both IL-10 and IFN-γ was increased in spleen CD8^+ ^cells (Figure 8B) and the expression of IFN-γ was also increased in the CD8^+^CD28^+ ^T cell population (Figure 8C). The proliferative response to the polyclonal mitogen Concanavalin A (ConA) was higher for CD8^+ ^cells than for CD4^+ ^cells in both groups and high-fat diet resulted in a further enhancement of the CD8^+ ^cell proliferative response (Figure 9C).

At 8 weeks IL-10 expression remained elevated in CD4^+ ^spleen cells from the high-fat group, but IFN-γ was now more frequently expressed than IL-10 by CD4^+ ^cells in both groups (Figure 8D). We did not detect differences in CD4^+^CD25^+^FoxP3^+ ^(2.3 ± 0.3% vs. 2.1 ± 0.6% of CD4^+ ^cells, n.s.) or CD4^+^CD28^+ ^IFN-γ^+ ^(27.7 ± 3.9% vs. 27.4 ± 4.1% of CD4^+ ^cells, n.s.). The CD8^+ ^expression of IFN-γ was higher than at 4 weeks, but there was no longer any difference between the groups (Figure 8E and 8F). However, the proliferative response of CD8^+ ^cells to ConA remained elevated in the high-fat group (Figure 9D). In order to elucidate this discrepancy of CD8^+ ^T cell cytokine production and proliferation we performed additional analyses studying the association between activation of DNA synthesis, IL-10 and IFN-γ expression in spleen CD8 T cells isolated from mice exposed to high-fat-diet. Interestingly, the results demonstrated a strong association between DNA synthesis and IFN-γ expression (r = 0.755, *p *= 0.005) along with a strong inverse association between DNA synthesis and IL-10 expression (r = -0.72, *p *= 0.008). These results suggest the induction of two distinct CD8 T cell phenotypes, one proliferative and pro-inflammatory and one quiescent and anti-inflammatory. To further asses the effect of hypercholesterolemia on the balance between Th1 and Th2 cells we analyzed the release of IFN-γ, IL-4 and IL-10 from cultured splenocytes exposed to ConA. An increased capacity to express IL-4 was only observed in mice given a high-fat diet for 4 weeks (table 1), whereas no effects were seen on ConA-induced IFN-γ and IL-10 secretion at any time point.

**Table 1:** Cytokine production of stimulated splenocytes

	IFN-γ (pg/ml)		IL-4 (pg/ml)		IL-10 (pg/ml)	
	Chow	HFD	Chow	HFD	Chow	HFD
4 weeks	620.8 ± 395.9	898.6 ± 784.3	8.5 ± 2.9	13.0 ± 3.4	83.3 ± 49.0	95.8 ± 80.0
*P*	n.s		**		n.s	
8 weeks	1052.0 ± 803.8	574.6 ± 382.5	8.3 ± 2.8	8.8 ± 2.4	75.6 ± 28.9	82.7 ± 43.5
*P*	n.s		n.s		n.s	

Concentration of IL-4, IL-10 and IFN-γ in cell supernatant of conA stimulated splenocytes from mice fed chow or high fat diet for 4 or 8 weeks. **P < 0.01.

### Antibody levels in plasma and atherosclerotic lesions

**Figure 10:**
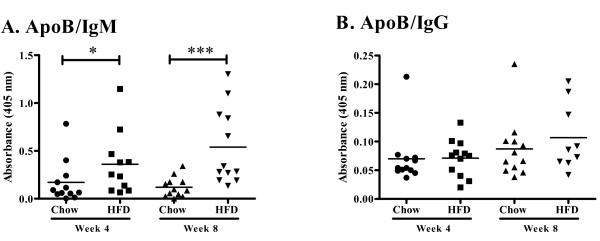
**Antibody titer of anti-ApoB IgM/IgG immune complexes**. Plasma antibody titer of (A) anti-ApoB IgM and (B) anti-ApoB IgG. *P < 0.05, ***P < 0.001.

**Figure 11:**
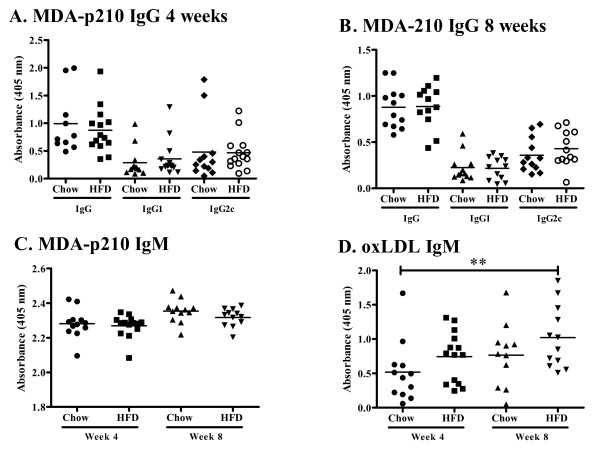
**Antibody titer of anti-MDA-p210 IgM, IgG, IgG1 and IgG2c and anti-oxLDL IgM**. Plasma antibody titer of anti-MDA-p210 IgG, IgG1 and IgG2c at (A) 4 and (B) 8 weeks, (C) anti-MDA-p210 IgM and (D) anti-oxLDL IgM. **P < 0.01.

**Figure 12:**
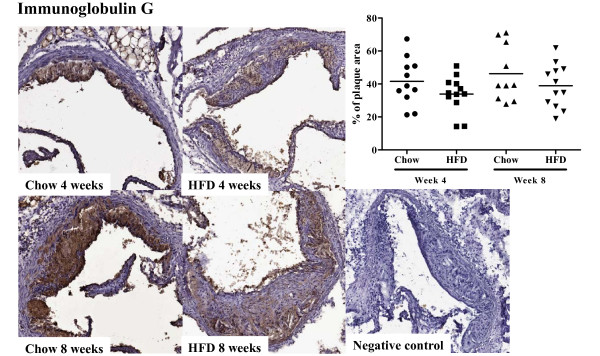
**Immunoglobulin G deposition in the aortic root**. Immunohistochemical staining and quantification of the fraction of immunoglobulin G (IgG) in aortic root sections from mice fed chow or high fat diet at 4 and 8 weeks respectively.

**Figure 13:**
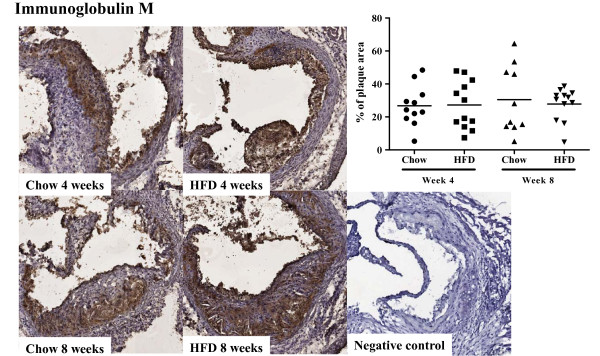
**Immunoglobulin M deposition in the aortic root**. Immunohistochemical staining and quantification of the fraction of immunoglobulin M (IgM) in aortic root sections from mice fed chow or high fat diet at 4 and 8 weeks respectively.

To assess the effect of increased plasma cholesterol levels on humoral immunity we determined plasma levels of apo B/IgM and IgG immune complexes, as well as autoantibodies against oxidized LDL and the oxidized LDL antigen MDA-p210 (the 3136-3155 amino acid sequence of apo B modified by malondialdehyde). High-fat diet increased the level of plasma apo B/IgM immune complexes as compared to chow (0.36 ± 0.33 vs. 0.17 ± 0.22 absorbance units at 4 weeks, p < 0.05 and 0.54 ± 0.40 vs. 0.12 ± 0.10 absorbance units at 8 weeks, p < 0.005, Figure 10A), whereas the level of apo B/IgG immune complexes were low and did not differ between the groups (Figure 10B). Plasma levels of IgM against oxidized LDL and MDA-p210 as well as IgG against MDA-p210 did not change in response to increased hypercholesterolemia (Figure 11A-D) while the IgM anti-oxLDL titer of mice fed high fat diet for 8 weeks was markedly increased compared to mice fed chow diet for 4 weeks (Figure 11D). The IgG, IgG1 and IgG2c antibody plasma concentration against oxLDL was below detection limit (data not shown) and there was also no effect on the MDA-p210 IgG1/IgG2a ratio (data not shown). To determine if plaque size and oxLDL accumulation affected the deposition of autoantibodies we stained aortic root lesions for presence of IgM and IgG antibodies. The fraction of IgM or IgG antibodies was not different in plaques from high-fat diet-fed *Apoe^-/- ^*mice compared to plaques from chow-fed *Apoe^-/- ^*mice (Figures 12 and 13) at any time point. Since mice fed high fat diet develop larger plaques than chow fed mice, this suggests a reciprocal relationship between autoantibody deposition and plaque size.

## Discussion

It is well established that adaptive immune responses induced by hypercholesterolemia play an important role in the development of atherosclerosis [2,3], but the pathways involved remain to be fully characterized. In the present study we assessed the response of CD4^+ ^and CD8^+ ^T cells, as well as humoral immunity, to a high-fat diet. At 4 weeks the immune response to an aggravated hypercholesterolemia was found to involve expression of IFN-γ by CD8^+^CD28^+ ^T cells in plaque-draining lymph nodes and in the spleen. At the latter location this pro-inflammatory response was counterbalanced by a concomitant release of IL-10 from both CD4^+ ^and CD8^+ ^T cells, whereas no induction of IL-10 occurred in plaque-draining lymph nodes. However, following an extended 8 weeks of high-fat diet activation of CD4^+ ^T cells could be observed also in draining lymph nodes. As opposed to the CD4^+ ^T cells in the spleen that remained in a phenotype characterized by expression of IL-10, the mediastinal lymph node CD4^+ ^T cells by then had differentiated into IFN-γ secreting Th1 cells. Our findings imply that when antigens generated in a hypercholesterolemic state are acquired systemically and presented to T cells in the spleen this results in differentiation of CD4^+ ^T cells into IL-10 producing Th2 cells. This notion is in accordance with previous studies by Zhou and coworkers demonstrating that diet-induced hypercholesterolemia is associated with a switch in oxidized LDL-specific IgG towards a Th2 isotype [27]. Our observation of an increased capacity of splenocytes from mice given high-fat diet to secrete IL-4 is also in accordance with a hypercholesterolemia-dependent shift towards Th2. In contrast, when antigens generated by hypercholesterolemia are acquired in atherosclerotic lesions and presented to T cells in draining lymph nodes this results in activation of Th1 cells. The reason why antigens generated by hypercholesterolemia activates a Th2 response systemically but a Th1 immune response when acquired by antigen presenting cells (APC) in atherosclerotic lesions remains to be elucidated. One possibility could be that the pro-inflammatory environment of the atherosclerotic plaque activates APC to express co-stimulatory molecules and cytokines in a manner that subsequently primes naïve T cells in draining lymph nodes to Th1 differentiation. Supporting this notion, dendritic cells pulsed with oxLDL aggravate atherosclerosis or are atheroprotective depending on the priming environment. Habets *et al*. used infused DCs pulsed with copper-oxidized LDL into *LDLr^-/- ^*hosts which reduced carotid lesion size and increased plaque stability [28]. On the contrary, MDA-LDL pulsed DCs aggravated atherosclerosis in *Apoe^-/- ^*mice [29]. Interestingly, immunization with MDA-LDL in complete Freunds adjuvant is atheroprotective [30], indicating that *ex vivo *primed DCs generate a pro-inflammatory response whereas in vivo primed DCs are anti-inflammatory. Gautier *et al*. reported that DCs in *Apoe^-/- ^*and *LDLr^-/- ^*mice are Th1 and Th17 disposed, while removal of DCs increase hypercholesterolemia, further supporting the dual role of DCs in atherosclerosis [31]. Since the capability of DCs to prime CD4^+ ^T cells is equivalent in normo- and hypercholesterolemic mice [32], DCs may initiate the Th1 priming associated with aggravated atherosclerosis as well as the Th2 response present in splenocytes of mice given high fat diet. It is also possible that Th1 priming of CD4^+ ^T cells in the mediastinal lymph node could be aided by the IFN-γ secreting CD8^+ ^CD28^+ ^T cells that develop in draining lymph nodes prior to the occurrence of CD4^+ ^Th1 cells.

An unexpected finding in the present study was that the initial pro-inflammatory response to hypercholesterolemia was started by CD8^+ ^T cells and not by CD4^+ ^T cells. The role of CD8^+ ^T cells in atherosclerosis has not been extensively studied. CD8^+ ^T cells are present in atherosclerotic lesions of *Apoe^-/- ^*mice, but are less frequent than CD4^+ ^T cells [33]. However, in advanced human lesions the CD8^+ ^T cells may account for up to 50% of the entire leukocyte population [34]. Targeting of a CD8^+ ^T cell immune response against arterial smooth muscle cells has been shown to markedly aggravate atherosclerosis in *Apoe^-/- ^*mice [35]. CD8^+ ^T cells have also been implicated in the accelerated atherosclerosis that occurs in patients with autoimmune diseases such as systemic lupus erythematosus [36]. In a study by Elhage and coworkers [37] CD8 deficiency did not influence atherosclerosis development in *Apoe^-/- ^*mice. However, in the same study it was unexpectedly found that deficiency of CD4 increased plaque formation. The authors concluded that this could be due to the expansion of CD8^+ ^T cell population that characterizes CD4^-/- ^mice.

Our findings do not permit any clear conclusion regarding the antigen(s) responsible for hypercholesterolemia-induced immune activation. It is generally assumed that oxidized LDL is a dominant autoantigen generated by hypercholesterolemia [38]. In agreement with previous studies oxidized LDL IgG and IgM autoantibodies were present in chow-fed *Apoe^-/- ^*mice [25] and the levels did not change significantly in response to high-fat diet. In contrast, mice fed a high-fat diet were found to have higher levels of apo B/IgM immune complexes in the circulation which could reflect an increase in the amount of circulating oxidized LDL as well as an enhanced generation of anti-oxidized LDL IgM. There was however no change in the anti-oxLDL IgG1/IgG2c ratio. This observation is in contrast to studies by Zhou *et al*. [27] demonstrating a shift towards Th2 isotype anti-oxidized LDL IgG in severe hypercholesterolemia. The reason for this discrepancy remains to be clarified but may involve the difference in antigens used in the ELISAs. However, the observation that high-fat diet increased the capacity of splenocytes to secrete IL-4 suggests that hypercholesterolemia stimulated a shift towards Th2 also in the present study. In order to further develop the assay, it would be interesting to determine if the origin of IL-4 production is CD4^+ ^or CD8^+ ^T cell specific. An important limitation that should be kept in mind when interpreting the present findings is that mouse models of atherosclerosis may not be entirely representative of the pathophysiology of human atherosclerosisis [39]. Moreover, it is also unlikely that the sudden increase in plasma cholesterol levels observed in response to high fat diet in the present study would occur in humans. Nevertheless, in spite of these limitations, our findings provide support for a role of CD8^+ ^T cells in immune responses activated by hypercholesterolemia.

The accumulation of oxidized LDL in atherosclerotic lesions was correlated to lesion size. In spite of this there were no signs of activation of CD4^+ ^T cells in draining lymph nodes 4 weeks after diet change, suggesting a lack of reactivity against extracellular antigens such as oxidized LDL. The observation of increased IFN-γ expression in CD8^+^CD28^+ ^T cells instead point to an initial immune response against cell antigens. Although immune responses against apoptotic cells generally are tolerogenic and inhibit atherosclerosis [40] it can not be excluded that this represents an immune response against damaged vascular cells.

## Conclusions

In conclusion the present observations demonstrate that hypercholesterolemia leads to activation of an unopposed Th1 immune response in lymph nodes draining atherosclerotic plaques, whereas this response in the spleen is balanced by a concomitant activation of IL-10 expressing Th2 cells. They also show that CD8^+ ^T cells may have an equally important role as CD4^+ ^T cells in hypercholesterolemia-induced immune activation implicating generation of cellular autoantigens.

## Authors' contributions

DK carried out the cell work, flow cytometry and data analysis. OHR participated in the cell work and flow cytometry. KEB participated in titration of assays and study design. JP carried out the immunohistochemistry. MW participated in the proliferation assays. HB, GNF and JN participated in the design and coordination of the study and JN wrote the manuscript. All authors read and approved the final manuscript.
